# Preoxygenation with high-flow nasal oxygen at various flow rates in elective surgical patients: a prospective, randomised, single-blind clinical trial

**DOI:** 10.1016/j.bja.2025.11.017

**Published:** 2025-12-18

**Authors:** Albin Sjöblom, Frida Hoffman, Magnus Hedberg, Ida-Maria Forsberg, Malin Jonsson Fagerlund

**Affiliations:** 1 Karolinska University Hospital, Stockholm, Sweden; 2 Karolinska Institutet, Stockholm, Sweden

**Keywords:** airway management, apnoea, high-flow nasal oxygen, hypercarbia, oxygenation, preoxygenation, tracheal intubation

## Abstract

**Background:**

High-flow nasal oxygen (HFNO) is increasingly used for preoxygenation. Previous studies of preoxygenation have explored flow rates below 60 L min^−1^. This study aimed to compare different HFNO flow rates during preoxygenation to identify the most effective flow rate for clinical use.

**Methods:**

This randomised trial included patients aged 18–84 yr of ASA physical status 1–3 scheduled for elective surgery. Preoxygenation was conducted using HFNO at randomised flow rates of 45, 70, or 95 L min^−1^. After preoxygenation, patients rated their level of preoxygenation-related discomfort. HFNO was discontinued at apnoea start. After intubation, apnoea was maintained until Spo_2_ reached 93%. Primary outcome was apnoea time until Spo_2_=93%. Secondary outcomes were Pao_2_ levels during preoxygenation and preoxygenation-related discomfort.

**Results:**

Of 72 participants included in the final analysis, baseline characteristics were similar and the mean duration of preoxygenation was 250 s in all three groups. Apnoea times did not differ, with median durations of 472, 523, and 483 s for 45, 70, and 95 L min^−1^, respectively (*P*=0.59). At apnoea onset, 95 L min^−1^ produced higher Pao_2_ than 45 L min^−1^. No other differences in Pao_2_ were observed during preoxygenation. The level of discomfort was lower for 45 L min^−1^ compared with 70 and 95 L min^−1^.

**Conclusions:**

There was no difference in the safe apnoea time between preoxygenation with the three flow rates. As 45 L min^−1^ generated the least discomfort while providing preoxygenation effectiveness comparable with higher flow rates, increasing preoxygenation flow rate from 45 to 70 L min^−1^ will likely not prolong safe apnoea time.

**Clinical trial registration:**

NCT 06736132.

**Editor’s key points**

• Use of high-flow nasal oxygen (HFNO) can enhance the effectiveness of preoxygenation, as recommended in recent guidelines.
• In a comparison of different HFNO flow rates during preoxygenation to identify the most effective flow rate for clinical use, this randomised study found no differences in safe apnoea time at flow rates of 45, 70, or 95 L min^−1^.
• Thus the lower flow rate of 45 L min^−1^ was as effective as higher flow rates, so increasing above this will likely not prolong safe apnoea time.

Preoxygenation before induction of general anaesthesia is a standard procedure aimed at maximising oxygen reserves to prolong the time until desaturation during anaesthesia-induced apnoea. Because of challenges in reliably predicting difficult airway scenarios, current guidelines recommend preoxygenation in all patients before induction of anaesthesia.^1^, ^2^, ^3^

Traditionally, preoxygenation has been performed using a tight-fitting facemask delivering oxygen 100%. During the past decade, high-flow nasal oxygen (HFNO) has been introduced for preoxygenation, also creating a seamless transition into apnoeic oxygenation after induction of anaesthesia.^4^^,^^5^ The technique has proved efficient in a range of clinical situations requiring rapid sequence induction, such as anaesthesia for emergency surgery patients, trauma patients, the critically ill, and during obstetric anaesthesia.^6^, ^7^, ^8^, ^9^, ^10^, ^11^, ^12^ Moreover, in a recent systematic review and network meta-analysis, head-up preoxygenation with HFNO was superior to a tight-fitting facemask in terms of safe apnoea time.^13^ Additional suggested benefits of HFNO compared with traditional facemasks are improved patient comfort and increased ease of use for anaesthetists.^9^^,^^14^ Early studies of HFNO during apnoea indicated a ventilatory effect compared with historical data.^4^^,^^5^ More recent studies comparing different flow rates show that there is no flow-dependent wash out of CO_2_ during apnoea.^15^, ^16^, ^17^, ^18^

HFNO with a closed mouth can obtain end-tidal oxygen (ETO_2_) levels comparable with those achieved with a tight-fitting facemask, which is augmented with increased oxygen flow.^19^^,^^20^ Moreover, HFNO with closed-mouth breathing generates a flow-dependent linear positive end-expiratory pressure (PEEP) effect of ∼1 cm H_2_O of PEEP for every 10 L min^−1^ of oxygen flow.^20^^,^^21^ During preoxygenation, this can represent a clinically relevant advantage, as increased PEEP is associated with increased functional residual capacity (FRC), thereby increasing oxygen reserves.^21^^,^^22^ Increased safe apnoea time has been demonstrated using HFNO in combination with a mouthpiece rather than a tight-fitting facemask as well.^23^

Preoxygenation efficacy is normally evaluated using ETO_2_, however, it is the safe apnoea time that reflects the time with adequate blood oxygenation.^13^^,^^24^^,^^25^ Therefore, despite a high ETO_2_, the safe apnoea time can be reduced by factors such as a low FRC, critical illness, elevated metabolic rate, increased oxygen consumption, age, and increased BMI.

Safe apnoea time after preoxygenation with HFNO has not been thoroughly investigated. HFNO is increasingly used for preoxygenation and has proved to be a valuable tool in various clinical settings. However, previous studies investigating HFNO for preoxygenation have only used flow rates up to 60 L min^−1^, and it is not known whether higher flow rates can further enhance the effectiveness of preoxygenation. We assessed the effectiveness of preoxygenation with HFNO at flow rates of 45, 70, and 95 L min^−1^ in patients scheduled for elective surgery in order to identify the optimal flow rate for clinical use.

## Methods

### Study site and patient population

This prospective, randomised controlled trial was conducted at the Karolinska University Hospital, Stockholm, between January and June 2025. Approval was attained from the Swedish Medical Product Agency (2024-10-02, 5.1-2024-43332) and the Swedish Ethical Review Authority (2024-07-02, 2024-04565-01). The study adhered to the Declaration of Helsinki and is reported in accordance with the CONSORT statement. Before patient enrolment, the study was registered at clinicaltrials.gov (NCT06736132). All participants received oral and written study information and signed an informed consent form.

Patients scheduled for elective colorectal, urological or gynaecological surgery, aged 18–84 yr, BMI <35 kg m^−2^, and ASA physical status ≤3 were included in the study. Exclusion criteria were: (1) cardiac disease (ischaemic heart disease, heart failure [NYHA ≥2], current arrhythmias, pulmonary hypertension), (2) severe asthma, moderate to severe chronic obstructive pulmonary disease, (3) pregnancy, (4) smoker or former smoker who quit less than 1 yr before inclusion, (5) baseline oxygen saturation <95%, (6) nasal obstruction, (7) known or anticipated difficult airway, and (8) not capable of understanding study information and signing a written consent. Throughout the enrolment period, eligible patients were screened by the study team using the local surgical planning system.

### Randomisation

Participants were randomly allocated to flow rates of 45, 70 or 95 L min^−1^. Block randomisation was done by a member of the study group using sealed envelopes assigned in blocks of 15, in a 1:1:1 ratio. The allocated flow rate was blinded to the patient and medical staff, including the anaesthetist in charge, but not to the members of the study group.

### Study protocol

Before surgery, all participants received a radial arterial line. At arrival to the operating theatre, ECG, invasive arterial blood pressure, pulse oximetry, and train-of-four monitoring (Philips Intellivue X3, Amsterdam, the Netherlands) were applied. An i.v. line was secured, and a standard infusion of electrolyte solution was started. Participants were then placed supine, in a 10 degrees reverse Trendelenburg position. All participants received preoxygenation using HFNO at Fio_2_ 1.0. To generate flow rates up to 95 L min^−1^ oxygen was delivered from a Hamilton-C1 ventilator (Hamilton Medical AG, Bonaduz, Switzerland) to the Optiflow+ nasal cannula (Fisher & Paykel Healthcare, Auckland, New Zealand) via the Hamilton-HF90 breathing circuit (Hamilton Medical AG). A size medium Optiflow+ nasal cannula was used for all subjects.

In the operating theatre, preoxygenation was conducted for 3 min, and participants were instructed to try to breathe with their mouths closed. During the initial 30 s of preoxygenation, the oxygen flow rate was gradually increased from 20–30 L min^−1^ to the randomised target flow rate by a member of the study team. After ∼2.5 min, participants were asked to rate the level of preoxygenation-associated discomfort by pointing with a finger on a visual analogue scale (VAS) from 0 to 10 (0=no discomfort, 10=maximal discomfort) as explained before preoxygenation.

Anaesthesia was induced after 3 min of preoxygenation. All participants received a high-dose neuromuscular blocking drug, and no facemask ventilation was performed between anaesthesia induction and tracheal intubation. The drug regime used consisted of a fast-acting opioid (fentanyl, alfentanil or remifentanil), propofol, and rocuronium (1 mg kg^−1^ i.v.) given slowly to avoid haemodynamic instability. At apnoea onset (assessed visually by a member in the study team), oxygen flow was terminated. Tracheal intubation was thereafter performed using a videolaryngoscope (C-MAC, Karl Storz, Tuttlingen, Germany) when the anaesthetist assessed the conditions as appropriate, typically within 45 to 60 s after the administration of rocuronium. The correct placement of the tracheal tube was confirmed visually by the anaesthetist in charge and a member of the study team. The tracheal tube was secured, and the participant was left apnoeic with the tracheal tube open to the atmosphere until the peripheral oxygen saturation declined to 93%. During apnoea, anaesthesia was maintained with intermittent boluses of propofol and, if necessary, either bolus doses of fentanyl or infusion of remifentanil. Upon reaching an Spo_2_ of 93%, mechanical ventilation was commenced, and the protocol was terminated.

Vital parameters were measured continuously. Neuromuscular block was monitored, using train-of-four every third minute during apnoea. Arterial blood gases were collected before preoxygenation (baseline), once every minute during preoxygenation, at apnoea start, once every minute during apnoea and when apnoea was terminated. Arterial blood gases were analysed within 10 min of sampling (ABL90 Flex Plus, Radiometer Medical Aps, Brønshøj, Denmark). At the initiation of mechanical ventilation, end-tidal carbon dioxide (ETCO_2_) levels were measured during the first three breaths (FLOW-i, Maquet Critical Care AB, Solna, Sweden or Avance CS^2^, GE Healthcare, Waukesha, WI, USA). Preoxygenation time was defined as the time from the start of preoxygenation until the start of apnoea. Apnoea time was defined as the time from the start of apnoea until an Spo_2_ of 93% was reached.

### Outcomes

The primary outcome was safe apnoea time (time from apnoea start until a peripheral oxygen saturation of 93%). Secondary outcomes were: (1) Pao_2_ at 1 min of preoxygenation, (2) Pao_2_ at 2 min of preoxygenation, (3) Pao_2_ at start of apnoea, (4) proportion of patients tolerating 360 s of apnoea, and (5) preoxygenation-related discomfort. There were two exploratory outcomes; (1) ETCO_2_ –Paco_2_ difference at termination of apnoea, and (2) Paco_2_ changes during apnoea.

### Statistics

The recruitment of 21 participants in each group was estimated to have 80% power to reject the null hypothesis at a two-sided α of 0.05 for a difference of 60 s in time to desaturation, assuming a mean (standard deviation, sd) time to desaturation of 344 (75) s with HFNO at 50 L min^−1^ and equal sd across all groups.^23^ To accommodate incomplete outcomes and dropouts, we aimed to include 25 participants in each group (75 in total).

The primary outcome was analysed using the Kruskal–Wallis test. Secondary and exploratory outcomes were assessed using one-way analysis of variance (anova), Kruskal–Wallis test, Mann–Whitney *U*-test or Fisher’s exact test as appropriate for the type and distribution of data. *Post hoc* analyses were conducted using the Mann–Whitney *U*-test. Pairwise comparisons were analysed using paired *t*-test. The Shapiro–Wilk test was used to assess normality.

Continuous data were presented as mean (sd) or median (IQR [range]) as appropriate and categorical data as number (%). For *post hoc* pairwise comparisons, a Bonferroni-adjusted *P*<0.0167 was considered statistically significant. All tests were performed using SPSS Statistics® 29 (IBM®, Armonk, NY, USA).

## Results

**Fig 1:**
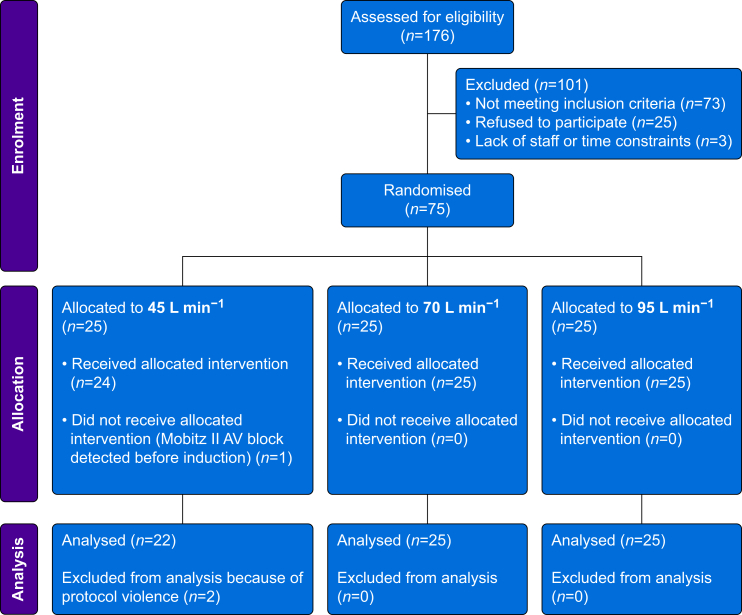
Study flow chart.

We included 75 participants who were randomly allocated to three groups of 25 with flow rates of 45, 70 or 95 L min^−1^. One participant was excluded before anaesthesia induction when Mobitz II atrioventricular block was detected. Two participants were excluded because of protocol violations, and data from 72 participants were analysed (Fig. 1). One participant did not tolerate the randomised flow rate of 95 L min^−1^ because of an itchy nose; therefore, the flow rate was gradually reduced to 70 L min^−1^ during preoxygenation. During data analysis, this participant remained in the 95 L min^−1^ group as per protocol. In the total cohort, 60% of participants were male with age, BMI, and waist circumference of 62 (12) yr, 25.2 (2.8) kg m^−2^, and 96 (10) cm, respectively. Most were classified as ASA physical status 2. There were no differences in baseline characteristics among the three groups (Table 1), and no differences in the duration of preoxygenation (Table 2).

**Table 1:** Participant characteristics. Values are mean (sd), mean (IQR [range]) or number (%) as appropriate. IQR, interquartile range; sd, standard deviation.

	45 L min^−1^ (*n*=22)	70 L min^−1^ (*n*=25)	95 L min^−1^ (*n*=25)
Sex			
Male	16 (73)	10 (40)	16 (64)
Female	6 (27)	15 (60)	9 (36)
Age (yr)	59 (55–65 [23–76])	64 (56–74 [29–83])	63 (56–72 [42–77])
BMI (kg m **^−^**^2^)	24.5 (2.5)	24.7 (3.0)	26.1 (2.9)
Waist circumference (cm)	95 (10)	90 (9)	99 (11)
ASA physical status			
1	3 (14)	4 (16)	4 (16)
2	19 (86)	16 (64)	20 (80)
3	0	5 (20)	1 (4)
Pulmonary comorbidity	1 (5)	1 (4)	2 (8)
Former smoker	4 (18)	5 (20)	4 (16)
Surgical procedure			
Colorectal	11 (50)	8 (32)	10 (40)
Urological/gynaecological	11 (50)	17 (68)	15 (60)

**Table 2:** Outcomes and between group comparisons. Data from induction of anaesthesia and primary and secondary outcomes. Values are mean (sd), median (IQR [range]) or number (%) as appropriate. IQR, interquartile range; sd, standard deviation.

	45 L min^−1^ (*n*=22)	70 L min^−1^ (*n*=25)	95 L min^−1^ (*n*=25)	*P*–value
Spo_2_ before preoxygenation (%)	100 (99–100 [98–100])	99 (98–100 [96–100])	100 (99–100 [95–100])	0.63
Duration of preoxygenation (s)	251 (23)	248 (21)	250 (20)	0.86
Closed mouth during preoxygenation				0.37
Yes	22 (100)	21 (84)	21 (84)	
Approximately 50% of the time	0	3 (12)	2 (8)	
To a small extent	0	1 (4)	2 (8)	
Paco_2_ at apnoea termination (kPa)	8.1 (0.2)	8.1 (0.19)	7.9 (0.19)	0.67
**Primary outcome**				
Duration of apnoea (s)	472 (402–670 [306–747])	523 (420–664 [305–759])	483 (375–605 [297–715])	0.59
**Secondary outcomes**				
Pao_2_ at 1 min of preoxygenation (kPa)	53.6 (9.1)	54.4 (10.6)	57.4 (14.3)	0.57
Pao_2_ at 2 min of preoxygenation (kPa)	64.0 (8.2)	64.4 (9.7)	67.7 (8.6)	0.30
Pao_2_ at apnoea start (kPa)	65.1 (62.4–69 [50.9–79.8])	67.3 (64.2–74.3 [56.9–79.1])	73.5 (67.4–77.6 [47.7–83.7])	0.01
Apnoea time >360 s; yes	19 (86)	24 (96)	20 (80)	0.22
Level of discomfort (0–10)	1 [0–2]	5 [2–5]	3 [1–5]	0.002

### Primary outcome

**Fig 2:**
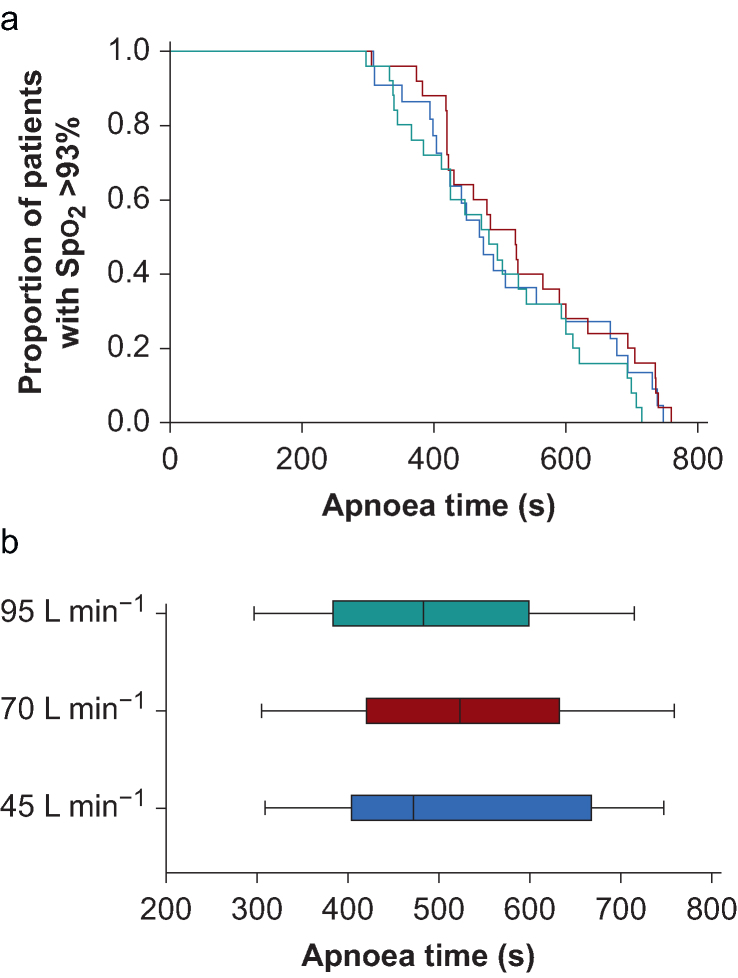
Apnoea times. (a) Proportion of patients with Spo_2_ >93% with high-flow nasal oxygen and flow rates of 45 L min **^−^**^1^ (), 70 L min**^−^**^1^ (), and 95 L min**^−^**^1^ (). No differences in the safe apnoea time were seen between the groups. (b) Median apnoea times after preoxygenation with high-flow nasal oxygen and flow rates of 45 L min**^−^**^1^, 70 L min**^−^**^1^, and 95 L min**^−^**^1^. Boxes represent the interquartile range, whiskers indicate the full range.

The safe apnoea time (from the start of apnoea until Spo_2_=93%) did not differ between groups, with median (IQR [range]) durations of 472 (402–670 [306–747]) s, 523 (420–664 [305–759]) s, and 483 (375–605 [297–715]) s for 45, 70, and 95 L min^−1^, respectively (*P*=0.59) (Fig. 2, Table 2).

### Secondary outcomes

Secondary outcomes are presented in Table 2. No differences were observed regarding Pao_2_ after 1 or 2 min of preoxygenation. At apnoea start, the 95 L min^−1^ group had higher Pao_2_ compared with the 45 L min^−1^ group (*P*=0.001). No differences were seen when comparing 95 with 70 L min^−1^ or 70 with 45 L min^−1^. Most participants tolerated 360 s of apnoea and no differences between groups were seen. Median (IQR [range]) level of preoxygenation-associated discomfort was lower in the 45 L min^−1^ group {1 (0–2 [0–5])} compared with both 70 L min^−1^ {5 (2–5 [0–10])} (*P*<0.001) and 95 L min^−1^ {3 (1–5 [0–8])} (*P*<0.001). No difference in discomfort was seen between 70 and 95 L min^−1^.

### Predefined exploratory outcomes

**Fig 3:**
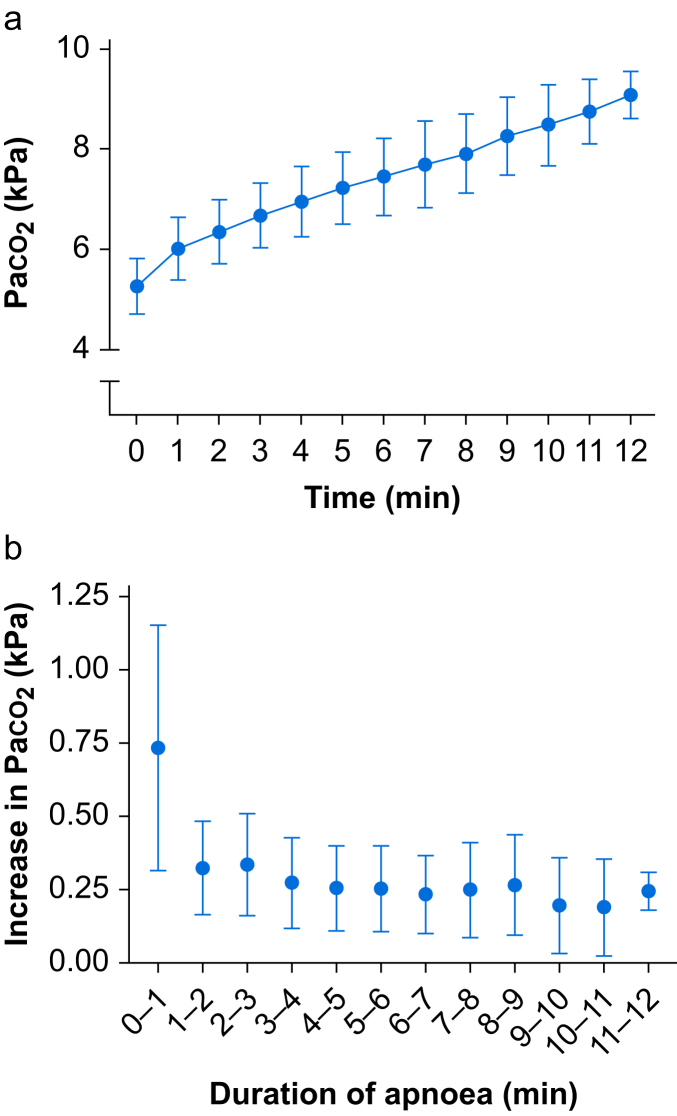
Increase in Paco_2_ during apnoea (a) and increase in Paco_2_ per minute during apnoea (b). Paco_2_ levels increased more during the first minute of apnoea (0.76 [0.41] kPa) compared with any other minute. The mean Paco_2_ increase per minute between minutes 1 and 12 ranged from 0.19 to 0.34 kPa m **^−^**^1^. Notably, the number of patients declined after 5 min of apnoea, as the protocol was terminated once Spo_2_ reached 93%. Data are presented as mean (standard deviation).

At termination of apnoea, Paco_2_ was higher than ETCO_2_ with a difference of 1.44 (0.57) kPa (*P*<0.001). The Paco_2_ continuously increased during apnoea in a nonlinear pattern, most pronounced during the first minute of apnoea, with an increase of 0.76 (0.41) kPa min^−1^ (Fig. 3). In the following minutes the increase in Paco_2_ ranged between 0.19 and 0.34 kPa min^−1^, thus significantly lower compared with the first minute. Any Paco_2_ value obtained during apnoea that was lower than an earlier value was deemed erroneous and was not included in analysis. This was seen in ∼20 Paco_2_ data points (3%), and these values were replaced using the last observation carried forward.

### Other exploratory outcomes

In 38 participants, where the respiratory rate was recorded at baseline and during preoxygenation, a reduction was observed compared with baseline, 16 (2) *vs* 11 (3), respectively (*P*<0.001). The decrease in respiratory rate did not differ between groups.

During preoxygenation, mean Paco_2_ for the whole cohort decreased from 5.0 (0.5) kPa at baseline to 4.8 (0.6) kPa after 3 min of preoxygenation (*P*=0.003).

Four participants in the 70 L min^−1^ group and four in the 95 L min^−1^ group were unable to breathe with a closed mouth during the entire preoxygenation. These participants had a safe apnoea time of 543 (154) s compared with 510 (133) s for the participants who maintained closed-mouth breathing throughout the entire preoxygenation (*P*=0.64).

## Discussion

In this randomised, single-blind study investigating preoxygenation using HFNO, we could not demonstrate any differences in the safe apnoea time between preoxygenation flow rates of 45 to 95 L min^−1^. Preoxygenation-associated discomfort was lower at 45 L min^−1^ compared with higher flow rates.

Previous studies have not investigated the use of HFNO for preoxygenation at flow rates >60 L min^−1^. Given the flow-dependent PEEP effect from HFNO, which has been shown to correlate with increased FRC,^21^^,^^22^ higher flow rates could potentially increase the oxygen reserve stored from preoxygenation and thereby prolong safe apnoea time. Despite this, we could not show any differences in safe apnoea time between groups. One explanation for this could be the increases in discomfort that were seen when increasing flow rates. As discomfort increases, the patients’ breathing pattern might be affected, leading to smaller tidal volumes limiting the potential benefits from the PEEP effect, while possibly also causing greater stress immediately before induction of anaesthesia.

In the present study, we demonstrated long safe apnoea times across all three flow rates, with median apnoea times ranging from 472 to 523 s. Despite the cohort being older and with slightly higher BMI, our safe apnoea times are considerably longer than those reported by Lyons and colleagues,^23^ who, using a similar study design, found a safe apnoea time of 344 s with HFNO at 50 L min^−1^. A possible reason for longer apnoea times in the present study is longer preoxygenation time; our mean duration of preoxygenation was near 4 min compared with near 3 min in the study by Lyons and colleagues.^23^ While Lyons and colleagues^23^ defined desaturation as an Spo_2_ of 92%, we used a similar cut-off of 93% based on our previous work and patient safety considerations.^7^, ^8^, ^9^

Compared with studies evaluating safe apnoea time after facemask preoxygenation, our results indicate longer apnoea times, even compared with studies using facemask preoxygenation combined with a 25-degree head-up position and continuous PEEP, both known to augment preoxygenation efficacy.^26^^,^^27^ However, preoxygenation with a facemask was not included in our study design and therefore a direct comparison is not possible.

While ETO_2_ is commonly used clinically to evaluate the effectiveness of preoxygenation, it does not consider other important factors also influencing time to desaturation such as FRC volume and oxygen metabolism. A high ETO_2_ after preoxygenation does not reflect the total volume of oxygen stored in the lungs, but rather indicates the degree of denitrogenation. In the event of an unanticipated difficult airway, tolerance of prolonged apnoea without desaturation is a major determinant of outcome. Therefore, our study focused on safe apnoea time as the primary outcome, defined as the duration from onset of apnoea to Spo_2_ 93%. This approach allowed us to consider all factors that affect preoxygenation effectiveness and the time until desaturation.

Patients generally reported low discomfort related to preoxygenation, although it was lower at 45 L min^−1^ compared with the higher flow rates. This low level of discomfort could help patients feel safer, potentially leading to reduced stress and lower oxygen demand. Additionally, HFNO for preoxygenation is well tolerated overall. In our cohort of 72 participants, only one could not tolerate a flow rate of 95 L min^−1^ because of an itchy nose, while all patients tolerated 45 and 70 L min^−1^. Regarding facemask preoxygenation, patient discomfort has been reported as greater compared with HFNO.^14^ HFNO might also provide a clinically superior alternative for preoxygenation in patients with anxiety, claustrophobia or in situations with facial injuries or malformations.

In line with previous findings,^5^ our results demonstrate a significant difference in Paco_2_ and ETCO_2_ at the end of apnoea, highlighting a limitation of ETCO_2_ in monitoring CO_2_ as it tends to underestimate Paco_2_. These differences are often explained by a progressive development of atelectasis during apnoea and increased shunt, although this has not been confirmed in clinical studies.^28^

When HFNO was first introduced for apnoeic oxygenation, data suggested that HFNO generated a flow-dependent wash out of carbon dioxide,^4^^,^^5^ when compared with historical data using low oxygen flow rates. Recent studies investigating flow rates ranging from 0 to 120 L min^−1^ have not identified flow-dependent difference in Paco_2_ increase while a flow-dependent CO_2_ washout effect has been dismissed.^15^, ^16^, ^17^, ^18^ We observed a similar increase in Paco_2_ to that reported in recent HFNO studies investigating apnoeic oxygenation despite the absence of oxygen flow. Thus, our results support the evidence that HFNO does not generate a flow-dependent increase in CO_2_ washout.

We demonstrate an almost three-fold increase in Paco_2_ during the first minute of apnoea compared with subsequent minutes, as also reported in other studies, although the temporal resolution has been lower and not always addressed.^5^^,^^28^^,^^29^ The mechanisms behind this initial high increase in CO_2_ are not fully understood, but could be attributable to equilibration between arterial and venous blood during apnoea, a higher metabolism as a result of light anaesthesia and noxious stimuli from tracheal intubation during the early phase, and the Haldane effect that releases CO_2_ from haemoglobin in response to high oxygen concentrations.

There are a few limitations to our study. It was a single-centre study at a centre with previous experience in the use of HFNO, which might limit the generalisability of the findings to less experienced centres. Additionally, although the inclusion criteria were relatively broad, we excluded patients with severe cardiopulmonary comorbidities, BMI >35 kg m^−2^ and those classified as ASA physical status 4 or 5. Therefore, our results may not be applicable to these populations.

## Conclusions

This randomised, single-blinded, study found no differences in the safe apnoea time when preoxygenation was performed with HFNO at flow rates of 45, 70, or 95 L min^−1^. At 45 L min^−1^, participants experienced the least discomfort while achieving preoxygenation effectiveness similar to the higher flow rates. Therefore, in populations similar to our cohort, increasing preoxygenation flow rate from 45 to 70 L min^−1^ will likely not prolong safe apnoea time.

## Authors’ contributions

Study design, patient recruitment, data collection: all authors

Data analysis: AS, MJF

Writing up of the first draft of the paper: AS

Final approval of the version to be published, agreement to be accountable for all aspects of the work: all authors

## Funding

Grants from ALF (973308), Swedish Research Council (2021-02787), Swedish Society of Medicine (999871, 1021440), Fraenkel Foundation, and Capio Foundations.

## Declaration of interest

MJF has received material support for academic studies from Fisher and Paykel Healthcare (Auckland, New Zealand). Fisher and Paykel had no influence on this study. The other authors declare that they have no conflicts of interest.
